# Supplementary material for: Seminoma and Embryonal Carcinoma Footprints Identified by Analysis of Integrated Genome-Wide Epigenetic and Expression Profiles of Germ Cell Cancer Cell Lines
Source: PLoS One. 2014 Jun 2;9(6):e98330. doi: 10.1371/journal.pone.0098330 (PMC4041891; doi:10.1371/journal.pone.0098330)
Supplement: File S1 — ZIP file containing DMRforPairs output for significant regions. Please start from the html files. (ZIP) [file pone.0098330.s008.zip › figures/25378.pdf]

RegionID: 25378, chr20:36148133−36149455−M_values

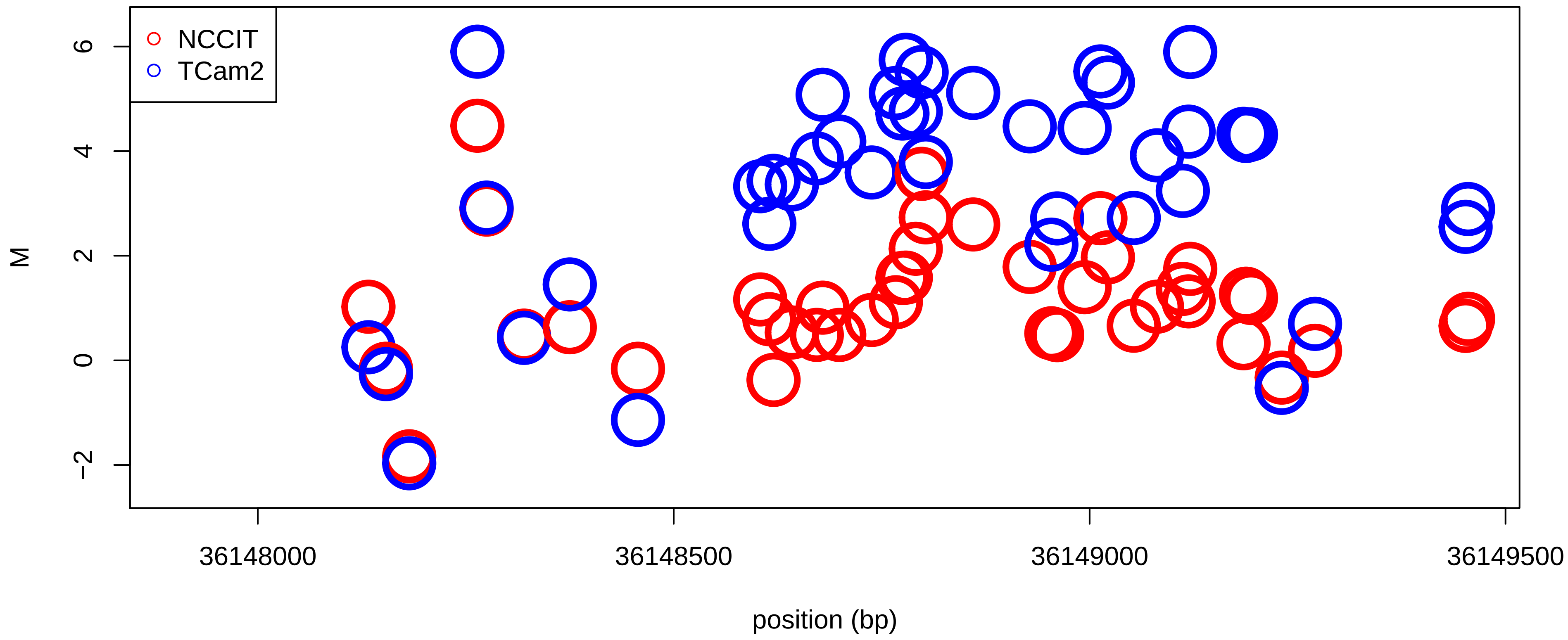

RegionID: 25378, chr20:36148133−36149455−Beta_values

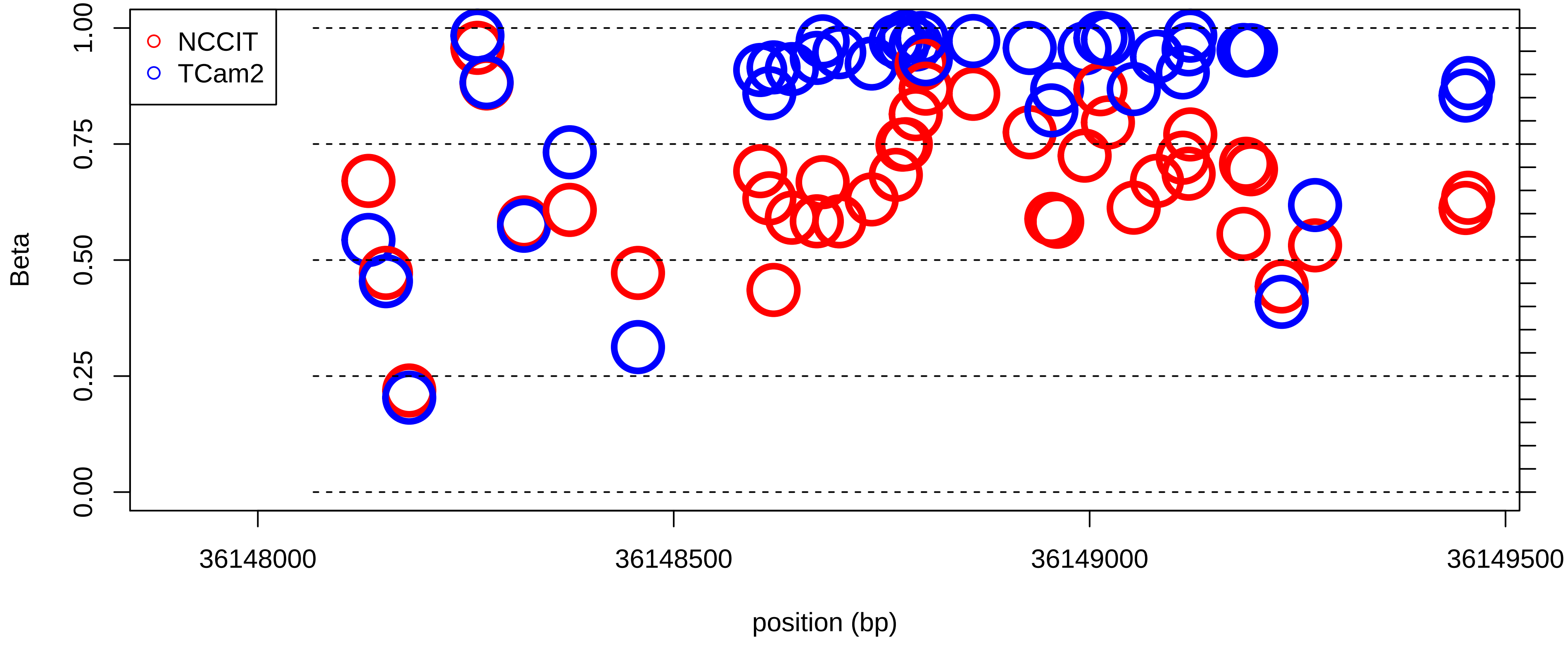

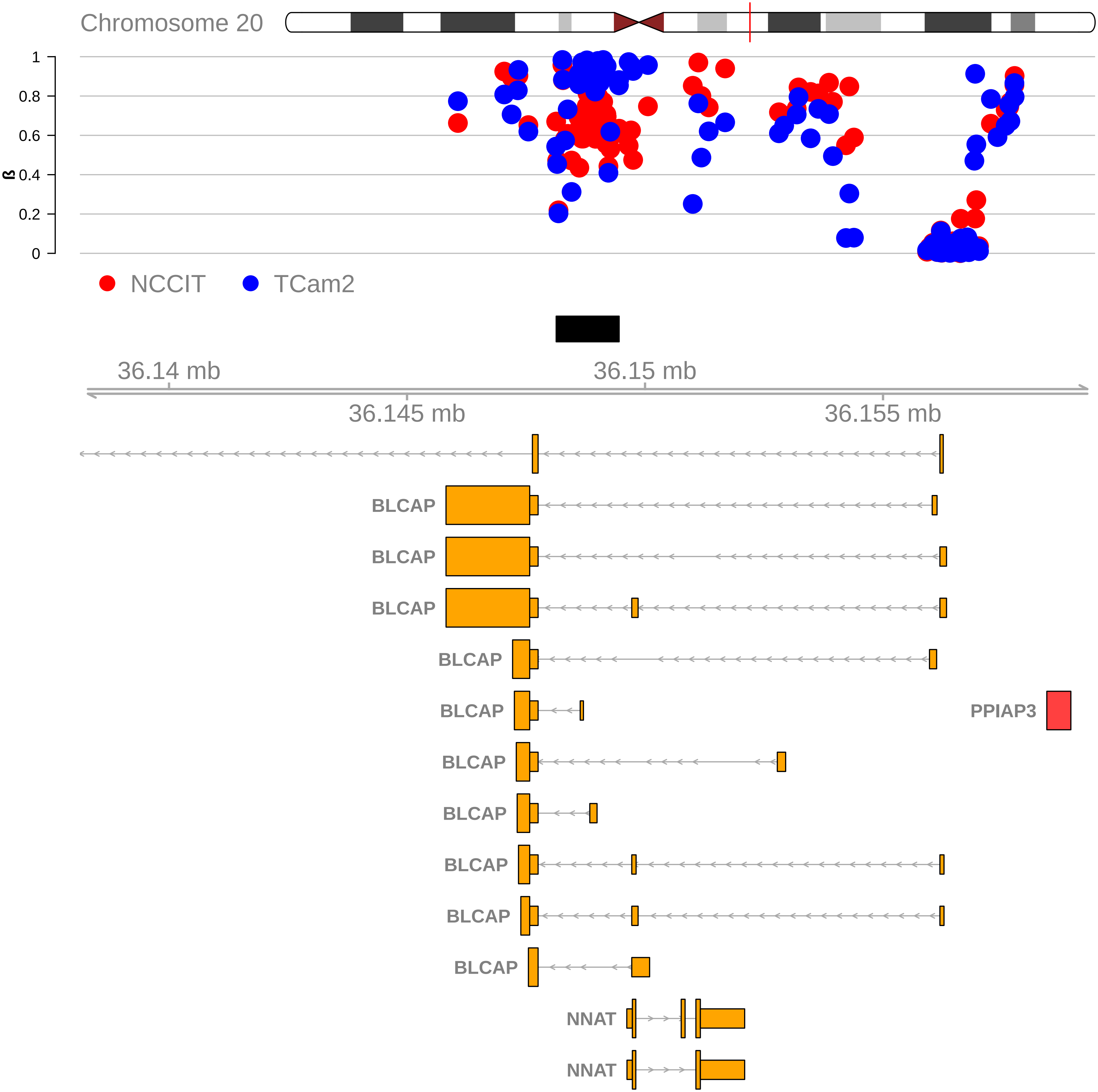
Chromosome 20
1
0.8
0.6
β 0.4
0.2
0
NCCIT
TCam2
36.14 mb
36.15 mb
36.145 mb
36.155 mb
BLCAP
BLCAP
BLCAP
BLCAP
BLCAP
PPIAP3
BLCAP
BLCAP
BLCAP
BLCAP
BLCAP
NNAT
NNAT
